# Building Climate Resilience in the Blue Nile/Abay Highlands: A Framework for Action

**DOI:** 10.3390/ijerph9020610

**Published:** 2012-02-16

**Authors:** Belay Simane, Benjamin F. Zaitchik, Desalegn Mesfin

**Affiliations:** 1 College of Development Studies, Addis Ababa University, Addis Ababa, Ethiopia; 2 Department of Earth and Planetary Sciences, Johns Hopkins University, Baltimore, MD 21218, USA; Email: zaitchik@jhu.edu; 3 Ethiopia Environmental Protection Authority, Addis Ababa, Ethiopia; Email: epa_ddg@ethionet.et

**Keywords:** adaptation, adaptive capacity, agroecosystem, climate change, innovation platforms, livelihood, resilience, vulnerability

## Abstract

Ethiopia has become warmer over the past century and human induced climate change will bring further warming over the next century at unprecedented rates. On the average, climate models show a tendency for higher mean annual rainfall and for wetter conditions, in particular during October, November and December, but there is much uncertainty about the future amount, distribution, timing and intensity of rainfall. Ethiopia’s low level of economic development, combined with its heavy dependence on agriculture and high population growth rate make the country particularly susceptible to the adverse effects of climate change. Nearly 90% of Ethiopia’s population lives in the Highlands, which include the critical Blue Nile (Abay) Highlands—a region that holds special importance due to its role in domestic agricultural production and international water resources. A five year study of climate vulnerability and adaptation strategies in communities of Choke Mountain, located in the center of the Abay Highlands, has informed a proposed framework for enhancing climate resilience in communities across the region. The framework is motivated by the critical need to enhance capacity to cope with climate change and, subsequently, to advance a carbon neutral and climate resilient economy in Ethiopia. The implicit hypothesis in applying a research framework for this effort is that science-based information, generated through improved understanding of impacts and vulnerabilities of local communities, can contribute to enhanced resilience strategies. We view adaptation to climate change in a wider context of changes, including, among others, market conditions, the political-institutional framework, and population dynamics. From a livelihood perspective, culture, historical settings, the diversity of income generation strategies, knowledge, and education are important factors that contribute to adaptive capacities. This paper reviews key findings of the Choke Mountain study, describes the principles of the climate resilience framework, and proposes an implementation strategy for climate resilient development to be applied in the Abay Highlands, with potential expansion to agricultural communities across the region and beyond.

## 1. Introduction

The 4th Report of the Intergovernmental Panel on Climate Change (IPCC), published in 2007, projects that the global temperature of the planet’s atmosphere will likely have increased 1.1 °C to 6.4 °C by the end of this century, relative to 1980–1999 baseline data. These temperature rises are linked to changes in precipitation patterns: some regions will see marked decreases in precipitation, while others will be affected by increases in total precipitation or changes in seasonality. An increase in the incidence and severity of extreme events (e.g., hurricanes and floods) has been reported as very likely as well [1].

Ethiopia has become warmer over the past century and human-induced climate change is expected to bring further warming at unprecedented rates over the next century. On the average, climate model projections included in the IPCC 4th Assessment Report show a tendency for increased rainfall, in particular during October, November and December. However, there was no clear model consensus in this prediction, with some models predicting a large increase and others a significant decrease in mean annual precipitation. Beyond the matter of changes in average annual precipitation, there is much uncertainty about the future distribution, timing and intensity of rainfall in the country [1]. Ethiopia’s low level of economic development combined with its heavy dependence on rain-fed agriculture, which is sensitive to climate change, and its high population growth rate make the country particularly exposed to climate change, and, due to low adaptive capacity, highly vulnerable to the adverse impacts of these changes [2,3]. Intense pressure on the country’s soil, water, and biodiversity resources from population growth and inappropriate traditional farming and management practices (extensive cultivation, overgrazing and deforestation and soil erosion) add to the climate change adaptation challenge [4,5,6]. 

The frequency and intensity of droughts has increased in recent years, severely affecting the livelihoods of millions of people [7,8]. At the same time, increases in floods have placed additional stress on social institutions and increased the vulnerability of households [7,8]. The drought conditions this year have most severely affected pastoralists and their animals, with the largest impacts in regions of northern Kenya, southern Ethiopia and Somalia where over 65% of the population are pastoralists. In some areas, up to 60% of the animals have already died from lack of water and pasture, depriving herders of their only source of income and food. Present estimates of vulnerable populations are 3.5 million in Kenya, 3.2 million in Ethiopia, 2.5 million in Somalia and 120,000 in Djibouti (UN report). These factors often interact with one another, resulting in a reinforcing cycle of “poverty, food insecurity and natural resources degradation trap” [2]. A recent study by the World Bank projects that unless steps to build resilience are effective, climate change will reduce Ethiopia’s GDP growth by between 0.5 and 2.5% each year [7].

In this paper *resilience* is defined as the ability of a community or ecosystem to resist, absorb, and recover from the effects of hazards in a timely and efficient manner, preserving or restoring its essential basic structures, functions and identity. We assume a resilient community is well-placed to perceive impacts of climate change properly and to minimize their effects and/or to recover quickly from any negative impacts, resulting in a similar or improved state as compared to before the hazard occurred. There are strong linkages between resilience and adaptive capacity. Consequently, resilience also varies greatly for different groups within a community. Adaptive capacity is defined as the potential of a system to adjust to climate change (including climate variability and extremes), to moderate potential damages, to take advantage of opportunities, or to cope with the consequences [9,10,11]. A system with strong *adaptive capacity* is expected to be *resilient* to climate variability, change, or extremes when they occur. One of the most important factors shaping the adaptive capacity of individuals, households and communities is their access to and control over natural, human, social, physical, and financial resources. Access to and control over the resources necessary for adaptation varies within countries, communities and even households. It is influenced by external factors such as policies, institutions and power structures. Adaptive capacity can vary over time based on changing conditions, and may differ in relation to particular hazards. 

Like elsewhere in Ethiopia, in the Abay Highlands there is misunderstanding and confusion about the impacts of climate change and how to deal with them, both amongst local development administrators and within the wider community. Institutions at different levels and communities with different adaptive capacities perceive the impact of climate change differently and each face different challenges in adapting to climate change effects. Additionally, experts have differing perspectives on where effort should be focused depending on the field of their specialization and the mandates of their institutions. This makes the planning process of building climate resilient economies complex, particularly when adaptation requires proactive investment in addition to reactive response [2,12,13]. 

The primary purpose of this paper is to present a framework for building resilience to climate change at a local level in the Blue Nile/Abay Highlands. Drawing on the findings of detailed studies in the communities of Choke Mountain, located at the center of the Blue Nile/Abay Highlands, it is argued that a *Community-based Ecosystem Approach* is the appropriate method both for assessing the impact of climate change and for engaging communities in sustainable adaptation options. The paper also introduces a new local institution—the Community-based Innovation Platform (IP)—that has been designed in accordance with this community-by-ecosystem organizing framework, and that is intended enhance community approaches to adaptation by applying a lens of ecological management to adaptation strategies at local to landscape scale. 

The paper proceeds as follows: Section 2 explains a framework for building resilience to climate change; Section 3 presents a review of findings from Choke Mountain that have informed the climate resilience framework; Section 4 summarizes opportunities and challenges for achieving climate resilient green economies in the Abay Highlands; Section 5 introduces an institutional mechanism for merging science, technology, and development in support of climate resilience, and Section 6 addresses conclusions and recommendations for up scaling the Choke experiences. 

## 2. A Framework for Building Resilience to Climate Change

In November 2011, the Government of Ethiopia initiated the Climate–Resilient Green Economy (CRGE) initiative to protect the country from the adverse effects of climate change and to build a green economy that will help realize its ambition of reaching middle-income status before 2025. Meeting this ambition requires understanding the causes and consequences of climate change as well as opportunities for climate change adaptation and mitigation. This is an extraordinarily complex problem, on account of the diversity of biophysical and socioeconomic conditions across the country. Ethiopia’s 80 million people are spread non-uniformly across approximately 1.13 million square kilometers, ranging from densely populated highlands (about 35% of total land area) to sparsely populated lowlands. Some 68 million rural people pursue predominantly agricultural livelihood strategies under conditions that vary widely in terms of moisture, temperature, disease, land quality and availability, remoteness from markets and services, *etc.* This translates to high variability in the farming systems and livelihoods pursued in different parts of the country. 

From the agricultural development perspective in the rural areas of Ethiopia, absolute and comparative advantages of different ecosystems and communities are fundamentally important frames for designing a climate resilient economy. Many studies of the impacts of different kinds of investments in east African highland production systems have shown the importance of biophysical and socioeconomic contexts for understanding impacts [11,13,14,15]. These studies clearly indicate that single, national-level agriculture adaptation strategies cannot work in a biophysically or socially diverse country. At the same time, there is clear benefit to leveraging adaptation knowledge and experience across communities, rather than attempting to develop a unique set of strategies for every community in the country. For this reason it is necessary to have some organizing framework for adaptation efforts that can be used to identify common adaptation contexts across diverse physical and cultural settings.

**Figure 1:**
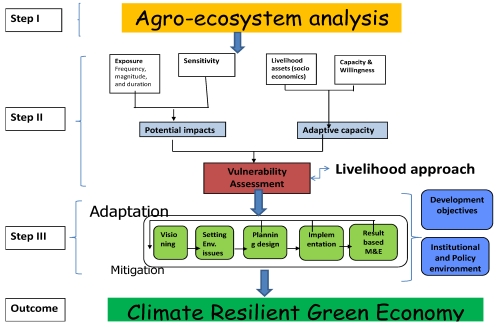
A conceptual framework for building resilience: *Community by Ecosystem-Based Approach*.

Motivated by this need for an organizing framework, a five year study of climate vulnerability and adaptation strategies was undertaken in the communities of Choke Mountain. The results of this study indicate that a *community by ecosystem-based approach* has significant potential for building climate resilience in communities across the region (Figure 1). Importantly, the framework places both human and natural systems at the center of analysis when considering vulnerability to climate change. The guiding principle is that impacts and vulnerabilities must be evaluated at the community by ecosystem level in order to generate science-based information for mitigation and adaptation. This information can be used to enhance coping capacity to climate change and subsequently to promote a carbon neutral and climate resilient economy in Ethiopia. The framework met with wide approval at a stakeholders’ workshop (Debre Markos, Ethiopia, 10–11 June 2011) [2] and at an expert meeting on climate resilience (Bahir Dar, Ethiopia, 9–10 July 2011). 

The framework begins with classification and analysis of major *agro-ecosystems* on the basis of ecology, soil, and farming systems. This provides the environmental context for climate risks and climate resilience. Next, potential *impacts* and the *vulnerability* of communities and sub-ecosystems within each agro-ecosystem must be evaluated. Methodologies of impact analysis are detailed elsewhere [16]. Based on our experience in the Choke Mountains and in other African countries, vulnerability assessment can be conducted following the Sustainable Livelihood Approach [13]. The approach places people, particularly rural poor people, the resources and *livelihood assets* (human, natural, financial, physical and social resources) that they have access to and use. The extent of their access to these assets is strongly influenced by their *vulnerability context*, which takes account of trends, shocks and seasonality. Access is also influenced by the prevailing social, institutional and political environment, which affects the ways in which people combine and use their assets to achieve their goals. These are their *livelihood strategies*. Adaptive capacity of society is used to describe the livelihood assets the communities have to plan, prepare for, facilitate and implement adaptation measures.

The third step of the framework posits that building climate resilience is possible through appropriate *adaptation and mitigation options* designed with consideration for local adaptive capacity and agro-ecosystem. Adaptation, for both ecosystems and human systems, is a process that requires the engagement of a wide range of stakeholders at multiple levels and in multiple sectors. It requires analysis of current exposure to climate shocks and stresses and model-based projections of future climate impacts. It demands an understanding of the existing vulnerability of individuals, households, and communities. With this information, adaptation strategies can be designed and implemented. Monitoring and evaluating the effectiveness of activities, as well as sharing knowledge and lessons learnt, are critical components of the process. The framework also recognizes that *policies and institutions* play a critical role in supporting or constraining people’s capacity to adapt to climate change.

A critical outcome of the research and consultations that informed this framework is that innovation platforms (IP) should be established in the Blue Nile/Abay Highlands. These innovation platforms, which are described further in Section 5 of the paper and in the report of the Debre Markos stakeholders’ workshop [2], will catalyze customization, dissemination, and uptake of proven climate resilient technologies and practices. It is critical that these centers be rooted in the region, in order to leverage community access and to take proper account of agro-ecosystems, livelihood contexts, existing policies and institutions, and development directions. 

The process described here recognizes that development interventions in agricultural regions are, generally speaking, inherently location-specific on account of the connection between economic development and the local natural resource base. In Ethiopia, however, there has been a long experience of top-down, technologically based solutions that have not given adequate consideration to the diversity of farming systems active in the country. These interventions have a poor record of success in terms of agricultural productivity or development gains, and in many cases a retreat to pre-intervention practices has been observed. In presenting a locally–oriented, empirically based framework for adaptation strategy that is endemic to the Ethiopian Highlands, we hope to establish a basis for better informed adaptation activities in the country.

## 3. Vulnerability & Adaptive Capacity: The Choke Mountain Experience

Building a climate resilient economy is about adapting effectively to climate change to minimize the potential damage and to maximize the potential benefits. A resilient community is well placed to manage hazards to minimize their effects and/or to recover quickly from any negative impacts, resulting in a similar or improved state as compared to before the hazard occurred. There are strong linkages between resilience and adaptive capacity; consequently, resilience also varies greatly for different groups within a community. One of the most important factors shaping the adaptive capacity of individuals, households and communities is their access to and control over natural, human, social, physical, and financial resources. Access to and control over the resources necessary for adaptation varies within countries, communities and even households.

**Figure 2:**
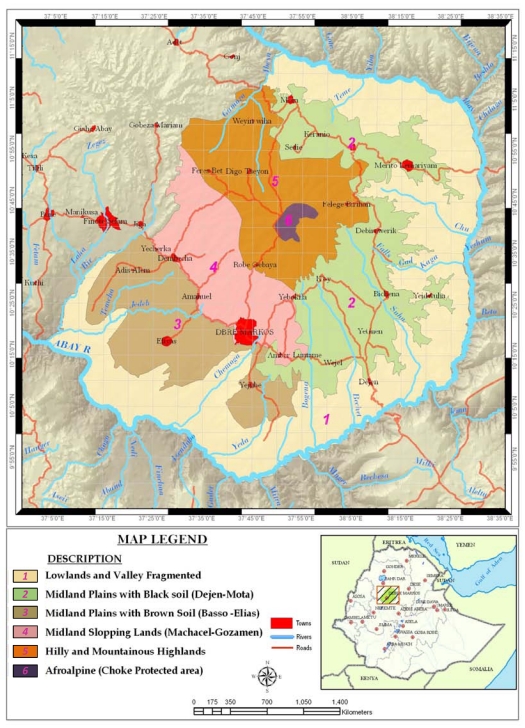
The six agro-ecosystems of the Choke Mountains watersheds.

The analysis and classification of Agro-ecosystems/Farming Systems in the Choke Mountain Watersheds was undertaken to support agricultural policy makers and development experts at Kebele, Woreda and Zonal levels in East Gojjam, the Amhara National Regional State [17]. The classification provides a lens for adaptation analysis that takes into account the geographical differentiation (climate, topography, soils, farming systems) as well as the socio-economic stratification of the agricultural sector of the study area [2,14,15]. Considering both sets of factors, we identified six major agroecosystems (AES) in the study area (Figure 2). 

These AES show a remarkable degree of differentiation in terms of constraints, opportunities, production orientation and socio-economic characteristics of farmers. All further analysis presented in this paper follow these classifications.

### 3.1. Farmers’ Perceptions

Adaptation to climate change requires that farmers first notice that the climate has changed, and then that they identify useful adaptation options and implement them [18,19]. People living in different agro-ecological systems are believed to perceive climate change differently, even when the systems are in close proximity to one another, due to contrasts in local climate impacts, as well as to differing socio-economic perspectives on these impacts. 

**Figure 3:**
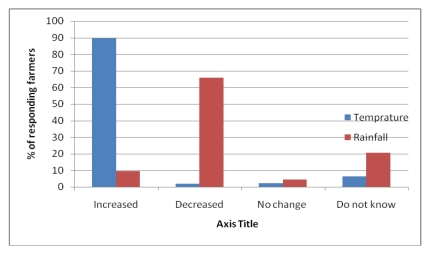
Farmers’ perception of long-term change in temperature and precipitation.

Analysis of perception of farmers across the agro-ecological systems of Choke Mountain showed no statistically significant variation in perception of temperature change: about 90% of the respondents perceived that the temperature has increased over the last 20 and 30 years (Figure 3). This is in agreement with scientifically observed temperature data of the area [20]. Perceptions of precipitation change, however, differed across agro-ecological zones, and this difference was significant at the 5% probability level. While 78% of farmers in the cold, sub-moist mid-highlands (AES 5) and warm sub-moist lowlands (AES 1) have perceived that there is a reduction in precipitation, only 35% of farmers in the cool and temperate midlands (AESs 2, 3 and 4) perceived such a reduction. Perceptions also differed regarding changes in the timing of rains and the frequency of extreme events. Averaged across agro-ecological systems, the majority of respondents (56%) perceived a change in the timing of rains, with rains coming earlier or later than expected, 34% observed increase in frequency of floods and droughts, and 10% claimed an increase in rainfall. 

These results are consistent with climate trends reported in Ethiopia’s First National Communications to the UNFCCC. As documented in that communication, climate change evidence is most clearly visible in temperature in Ethiopia, with an increasing trend in time (0.37 °C/decade). There is no clear trend in total annual rainfall observed at national level, but local changes in variability have been reported. These general patterns have held true for the Abay Highlands as well. Changes in timing and distribution of precipitation in the Abay Highlands are a subject of future research effort. 

In response to perceived changes in timing and variability of precipitation, farmers in the Choke Mountain Watersheds have tried to make some adjustments by shifting the cropping calendar, particularly in response to changes in the beginning and end of the rainy season. Many households have made adjustments in response to increased temperatures as well (e.g., changing crop species, timing of agronomic practices). Overall, 74.4% of sample households surveyed in the Choke Mountain study report that they have taken adjustment measures against climate change (Table 1). The number of farmers who have taken adjustment measures varied between AESs. For instance, in the cold sub-moist mid-highlands (AES 5) 90% of sample households have adjusted their cropping with the change in rainfall patterns. However, in the temperate moist mid-highlands (AES 3) only 58.3% of sample households have made adjustments. Farmers have adopted a wide range of response measures, including: reducing social spending in favor of saving (85% of respondents), storing grain (84%), reducing food consumption (72%), selling livestock (44%), inter-household transfers and loans (40%), selling forest products (19%), wage labor (8%), credit from merchants or money lenders (4%), migration in search of employment (3%), and sale of household assets (2%). 

**Table 1:** Farmers that have taken adjustment measures to climate change (%).

AESs	Yes (%)	No (%)	ANOVA test	
			F-value	Significance
Choke Mountain watersheds	74.4	25.6	4.45	0.075 *
AES 5 (Very cold sub-moist mid highlands )	69	31		
AES 4 (Cool sub-moist mid highlands)	85.7	14.3		
AES 3 (Temperate moist mid highlands )	58.3	41.7		
AES 2 (Cool -Moist Mid highlands )	85	15		
AES 1 (Warm sub-moist lowlands )	78.3	21.7		

* Significant at 10%.

### 3.2. Vulnerabilities and Adaptive Capacity by AES

**Figure 4:**
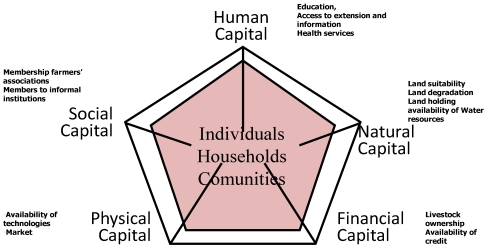
Conceptualising vulnerability: From adaptive capacities to adaptation.

Vulnerability assessment was conducted for the five major agro-ecosystems in the Choke Mountain Watersheds with extensive agricultural activity (AES 1–5). Data on 29 socio-economic, climatic and soil related variables were collected for communities across the five assessed agro-ecosystems, and, through regression analysis, 13 indicators were identified as explanatory of adaptive capacity at the community level (Figure 4). In this analysis, livelihood assets—the resources at the disposition of the households—were used as a proxy for adaptive capacity.

**Figure 5:**
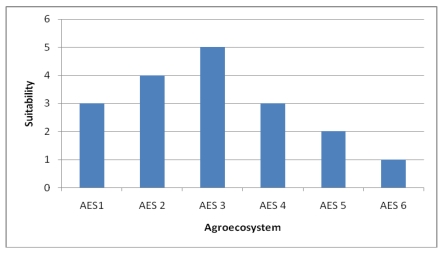
Land suitability of Choke Mountain watersheds by agroecosystems.

We identified four indicators related to **natural capital** that describe adaptive capacity, which explains vulnerability from a livelihood perspective. The first is *land suitability*, representing the suitability of different areas for crop production. This is based on the climate, soil and terrain conditions that are relevant to agricultural production (Figure 5). Suitability is then ranked on a scale from 1 (least suitable) to 5 (most suitable). We hypothesized that vulnerability increases with an increase in crop suitability, as household livelihoods are more at risk from substantial changes in climate. AES 1 is constrained with shallow soil depth, low fertility and sloping terrain. While AES 2 is only constrained with drainage, AES 3 in not constrained and found to be the most suitable land for agriculture. AES 4 is constrained with natural fertility due to leaching and acidity. AES 5 is constrained with all the parameters except drainage.

**Figure 6:**
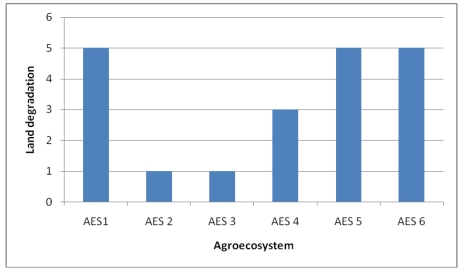
Human-induced land degradation of Choke Mountain watersheds by agroecosystems.

The second indicator is the severity of *human-induced degradation*, for which we used the FAO data. The severity of human-induced wind and water erosion is indicated by a combination of the degree and the relative extent of the degradation process. The erosion categories are classified into five major classes of degradation, from none to very severe. The hypothesis here is that the higher the human-induced soil degradation potential, the higher the vulnerability of the household. AES 1 and AES 5 are characterized by fragmented and steep slopes with the highest degradation rate (Figure 6). AES 2 and 3 have no visible soil erosion problems as they are the mountain toes. AES 4 is prone to moderate soil erosion. 

**Figure 7:**
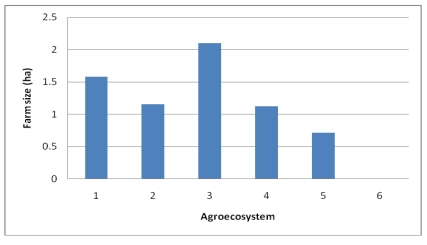
Average size of household farmlands of the Choke Mountains by agroecosystem.

The third indicator of natural capital is the *land holding* size suitable for crop and livestock production. From a livelihoods perspective, the hypothesis here is that the more land holding per household, the more volume the household can produce and the greater their flexibility to use diversified cropping systems. The average land holding size of the watershed is 1.4 ha (SD = 1.01, min = 0.06 and max = 8.25 ha) (Figure 7). AES 6 is the mountain summit with natural vegetation and is used commonly for grazing and as source fuel and construction wood.

**Figure 8:**
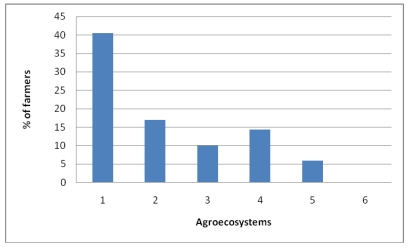
Percentage of farmers practicing irrigation.

The fourth indicator relating to natural capital is the availability of *water resources* (amount and distribution of rainfall irrigation potential) in the respective agroecosystems. The hypothesis is that the more water available in the landscape, the lower the vulnerability of the household. As presented in Figure 8, the use of irrigation by farmers in the whole Choke Mountain catchment is very low as compared with the available water resources (17.5%). Application of irrigation varies across agroecosystems. While 43.3% of the farmers in the Cold sub-moist mid-highlands (AES 3) are practicing irrigation, the numbers of households practicing irrigation in some other AES are very low, ranging from 5.6% in the very cold sub-moist mid-highlands (AES 5) to 17% in the cool-moist mid-highlands of Mahibere Berhan. In the Abay Gorges of Gelgelie and Kurar (AES 1) about 40.5% farmers are using some form of partial irrigation.

We included two indicators of **physical capital**, availability of technologies (improved seed, fertilizer, *etc.*) and accessibility to markets. From a livelihoods perspective, the hypothesis here is that both of these indicators are predictive of the potential for diverse household income sources. Proximity to markets is also indicative of the household’s access to other services. In the Choke Mountain Watersheds, on the average 75.2% of the sample surveys are applying chemical fertilizers for their crop production. These applications range from 0% in AES 5 to 97.9% in AES 2. Most farmers who make use of chemical fertilizer are not applying the recommended amount, on account of price, limited knowledge, and/or limitations in extension services. The number of farmers who have applied chemical fertilizers to enhance production of crops in the cool sub-moist mid-highlands (AES 4), temperate moist mid-highlands (AES 3) is also high (96.4%, 97.9% and 52.9% respectively).

**Figure 9:**
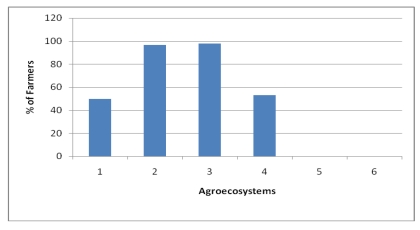
Percentage of farmers applying chemical fertilizer.

**Figure 10:**
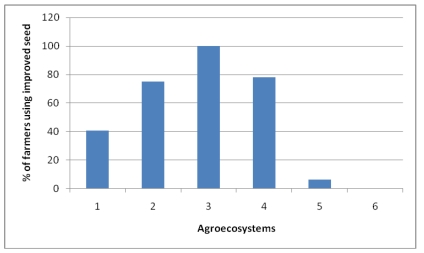
Percentage of farmers applying improved seeds in the Choke Mountains.

On the average, 63.6% of the farmers in the Choke Mountain use improved seeds (Figure 10). However, the use of improved seeds for crop agriculture varies with AES. For instance, in Temperate moist mid highlands of AES 3 and AES 2 all the respondents have used improved seed varieties whereas, in the very cold sub-moist mid highlands (AES 5) only 5.9% have done so. In general, utilization of improved seed varieties in the midland agro ecologies of cool sub-moist mid-highlands, cool moist mid-highlands, and temperate moist mid-highlands is higher than in the two extreme very cold sub-moist mid-highlands and warm sub-moist lowlands of Choke Mountain. Sixty-six percent of farmers in the area are also applying improved conservation measures, though these practices also vary significantly with AES (Table 2). 

**Table 2:** Farmers’ use of improved soil conservation practices for crop production.

AEZs	Soil conservation (%)	ANOVA test	
		F-value	Significance
Choke Mountain watersheds	66.7	1.89	0.09*
Very cold sub-moist mid highlands (AES 5)	67.6		
Cold sub-moist mid highlands (AES 4)	80.5		
Cool -Moist Mid highlands (AES 3)	17.5		
Temperate moist mid highlands (AES 2)	14.9		
Warm sub-moist lowlands (AES 1)	87.1		

* Significant at 10%.

We identified two indicators of **financial capital**, *livestock ownership* and *availability of credit*. From an adaptive capacity perspective we hypothesized that households with more livestock could cope with any disaster related to climate change at least for a short period by selling their livestock. The two indicators are not unrelated, as livestock holdings also open up access to credit, thus improving income diversification and access to technologies. We found no significant differences in terms these assets between AES.

We identified four indicators of **human capital**, *education*, *access to extension service*, *information* (knowledge about available adaptation options) and *health services*. From a livelihoods perspective, the hypothesis here is that the more educated, informed and healthy communities are, the more they can adapt proactively to climate change with the available natural and financial capitals. No significant differences were found in the human capital indicators between AES. 

For **social capital**, we found only one indicator, membership of households in *formal farmers’ associations*. However, as all households are members of informal institutions, we found no basis for quantifying differences in this term.

**Figure 11:**
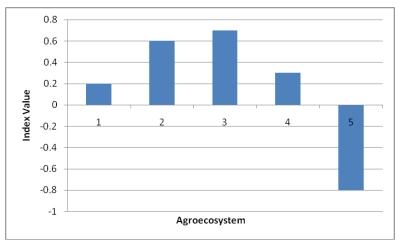
Adaptive capacity indices of the Choke Mountain watersheds.

Adaptive capacity analysis was conducted using access to and control over natural, human, social, physical, and financial resources [21,22]. Access to and control over the resources necessary for adaptation varies within communities in the different agroecosystems (Figure 11). While AES 3 and 2 exhibited relatively high adaptive capacities, AES 5 and 1 were found to have low adaptive capacity and consequently vulnerable to climate change impacts.

### 3.3. Farmers’ Participation in Climate Change Adaptation Efforts

There are growing opportunities for local level development projects targeted to adapt and manage climate change risks, supported by national and international investments. However, a major obstacle to integrating climate and development at community level stems from lack of capable local institutions to coordinate and lead local efforts. This is matched by a lack of active participation by local communities and lack of technically skilled manpower at the local level. In recognition of these limitations, a 5-step planning tool on *Local-Level Environment Action-Plan Development* has been developed to demonstrate and facilitate the preparation and implementation of environmental management action plan that are specific to a particular environmental problem or locality with the involvement of all stakeholders [23]. Following this approach, 21 farming communities in the Choke Mountain were organized and provided with about 25,000 USD each to develop their Local-level Environmental Action Plans (LEAP) and to implement climate change adaptation and biodiversity conservation projects through the support of Addis Ababa University and a Small Grants Program of the UNDP [2]. This investment provided an opportunity to evaluate participation in various climate resilience building activities organized at the community level.

A participation index analysis of farmers’ participation level in all watershed management activities verified that the vast majority of the farmers (96.4%) have participated in fewer than 60% of intended activities. Furthermore, over half of the farmers were found to participate in fewer than 40% percent of intended activities. Looking at participation in project stages, it was found that 51%, 43% and 1% of respondents participated in planning, implementation, and monitoring and evaluation (M&E) phases of the intervention, respectively. Regression results revealed that training, frequency of visit by extension workers, farmers’ perception about the benefit of participatory watershed management, and farmers’ perception of responsibility for watershed resource conservation have statistically significant and positive relationships with farmer participation. The presence of these community–base projects, in its own right, was found to increase community participation in natural resources governance, providing momentum for resilience building activities [24].

It should be noted that the Choke Mountain experience argues for a broad definition of what constitutes “adaptation technologies.” Technologies, in this context, include physical equipment, but they also include techniques, practical knowledge, or skills for performing a particular activity. Adaptation technologies can thus be defined as the application of technology in order to reduce the vulnerability, or enhance the resilience, of a natural or human system to the impacts of climate change. At the broadest level of classification, then, adaptation technologies can include hard technologies, such as irrigation technologies, fertilizer, and improved varieties of crops, as well as soft technologies such as crop rotation patterns, knowledge of varieties, and organizational capacity. A successful adaptation strategy will in general utilize both hard and soft technologies, which, rather than applied in a one-off activity, will involve the application of a combination of these technologies in an ongoing interactive process [25]. Population growth combined with new technology options and/or market opportunities can induce farmers to diversify and intensify systems. Depending on the natural resource base and management systems, intensification can either sustain and improve productivity over time, or degrade the natural resource base and therefore lower production potential over time.

## 4. Opportunities and Challenges

In application, research at the Choke Mountain Watersheds has indicated that there are a number of important opportunities and equally significant challenges for these communities as they transition to climate resilient economies. These opportunities and challenges are summarized below. 

### 4.1. Opportunities

- *Investment potential*: Even though communities in the Blue Nile/Abay highlands are relatively poor in their adaptive capacity, there are non-negligible livelihood assets and other resources available at the household and community levels that can be invested in improved adaptive capacity. The research confirms that it is possible to apply available adaptive capacities to sustainable adaptation actions, provided that investments are well planned and implemented with the full awareness and participation of community members.
- *Past experience with climate extremes*: Ethiopia is historically prone to extreme weather events. Rainfall is highly erratic, and most rain falls intensively, often as convective storms, with very high rainfall intensity and extreme spatial and temporal variability. Therefore there is a good opportunity to use past climate variability to inform future resilience strategies.
- *Application of Earth System Science information*: Earth System Science (ESS) is fundamental to advance our understanding of both natural and human systems. Institutions within Ethiopia and internationally are engaged in ESS studies relevant to the Blue Nile/Abay Highlands, and these efforts can be applied to informed climate adaptation in the region.
- *Integration of ESS to the adaptation dialogue*: Information from ESS and climate resilience analysis can be more effectively integrated to support policies and strategic decision support systems at the national and local level. Research at Choke Mountain has demonstrated that farmer perception and reactive adaptive actions are largely consistent with objective ESS analysis of recent climate variability in the region. This consistency suggests that there is great potential to inform proactive adaptation actions through consultative analysis of ESS projections relative to existing adaptive capacity at household and community level.

### 4.2. Challenges

- *Low investment rate and population pressure*: Ethiopia is one of the least developed countries in the world, even though the economy is reported to have grown by an average rate of 11 percent over the last five years. With a current population growth rate of about 2.8 percent per year, Ethiopia’s population is expected to reach 129 million by 2030 [26]. Because of poverty and high population pressure, which together result in extremely small landholdings, investment in improved land management is very low, leading to high susceptibility to adverse effects of climate variability and change. The natural resources base (land, water and biodiversity) is under intense pressure from population growth and inappropriate traditional farming and management practices.
- *Inherent limits on agricultural productivity*: Agriculture in the Blue Nile Highlands is mainly a subsistence, low input and output system. There are also widespread problems related to extensive cultivation, overgrazing and deforestation, and consequent soil erosion and soil fertility decline. Water scarcity, livestock feed shortages, and fuel wood needs are additional and significant pressures. The quality of land management has become an increasing matter of concern due to the additional stress of climate change. Land degradation problems are likely to be exacerbated by a rise in the frequency of extreme weather events.
- *Uncertainty in the climate forecast*: A major challenge for translating climate change assessments into adaptation policies and measures is persistent and deep uncertainty about future changes in climate conditions. For example, there is little consensus between different climate models regarding changes in precipitation amount, timing, and intensity. Uncertainty regarding future socio-economic states may be just as profound, yet climate vulnerability assessments commonly estimate the impacts of future climate changes based upon current social and economic conditions. This can lead to distorted perceptions of risk and appropriate adaptation responses.

## 5. Realizing Climate Resilience: The Role of Climate Innovation Platforms

The concept of Community and Ecosystem-based Adaptation in rural areas of Ethiopia encompasses a wide range of strategies at local and landscape scales, enabling communities to address climate change in an effective way. It is envisioned that ecosystem-based adaptation interventions will be developed and implemented by community-based IPs, and that they will be a component of a broader adaptation strategy that includes education, training, awareness rising, and the development of early warning systems and technology measures as required.

As described above, 21 targeted Community-Based Adaptation (CBA) development projects have been implemented in Choke Mountain watersheds over the last 5 years. The approach has centered on individual households within target micro-watersheds, with a focus on empowering vulnerable people with the knowledge, skills and resources they need to take action on the climate change adaptation strategies appropriate for their lives and livelihoods. In all projects, preservation of the natural resource base was taken as an entry point for planning adaptive actions [2]. The projects are rooted in a participatory, comprehensive analysis of the biophysical vulnerability that allows different groups—such as poor women or other marginalized people in the community—to identify targeted strategies based on their specific needs and priorities. 

**Figure 12:**
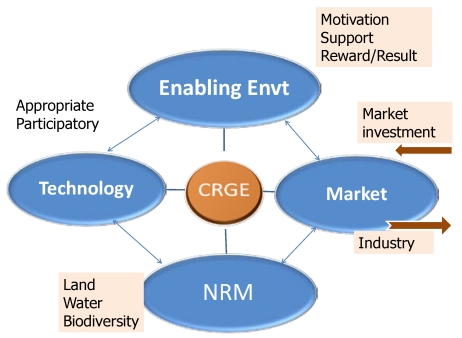
The four pillars of innovation platforms (NRM: Natural Resource Management). Following experience from Choke Mountain, all IP assessments will be approached on an agroecosystem by community basis.

While much has been learned through these projects, the sustainability of projects that take the natural resource base as an entry point has come into question. Interventions that are effective during the active project period, when external investments in adaptation capacity are made available, often fail to establish the link to markets that is required to sustain efforts after the project comes to a close. Based on this experience, participants have concluded that markets are a more appropriate entry and exit point for future resilience building efforts. This recognition has yielded a model that centers on the establishment and implementation of community-based IP, devoted to achieving a climate resilient green economy through dissemination and uptake of proven technologies and practices (Figure 12). As learned at Choke Mountain, an effective partnership is a necessary precondition to market-based technology transfer. The IP model also acknowledges that it is necessary to establish an enabling policy environment to make the partnership arrangement work legally. 

Inherent to the IP concept is the fact that innovation is driven by broad stakeholder involvement: farmers and their organizations, representatives of (national and international) research and extension, the private sector (input and output markets, food processing, transport, rural credit), local government (policy, subsidies, and rural credit), NGOs and others. These stakeholders must begin by analyzing challenges and limitations to livelihood improvement at the household and community level and by defining priority issues to address. Then, collectively, participants in an IP will identify and rigorously test proposed solutions. Finally, the IP is also responsible for implementing promising solutions, monitoring the implementation, and addressing problems or new challenges that arise in the course of time. 

A detailed description of the IP model is beyond the scope of the current paper, but is available elsewhere [2]. To summarize briefly, it should be emphasized that the IP model follows a systems approach that considers the entire agricultural production system, relevant value-chains, physical environment, and the interactions between them. Further, the IP model approaches community-based adaptation as a process that involves community empowerment, development and application of appropriate technologies and practices, and the establishment of vibrant market connectivity. This process requires time and investment, and it also requires deliberate engagement with communities in a manner that respects traditions but is, when necessary, freed from prevailing power dynamics as much as possible. IP sessions that engage only women from the community, for example, are found to yield insight on adaptation priorities and innovation opportunities that are not emphasized in mixed-gender settings. In this regard, IP activities have been informed by the experience of similar development-oriented community interventions elsewhere in Africa (as described in [2]).

In the coming years, a total of 30 Innovation Platforms (three IP per agroecosystem) will be established in the Blue Nile Highlands. Modalities for implementation and funding have been developed by the Ethiopian Environmental Protection Authority in close consultation with academics and stakeholder groups. Experience at Choke Mountain has provided a strong foundation for this effort, but it will be critical to maintain active analysis and a flexible approach to implementation as the IP experiment moves forward. 

The major activities of these IPs focus on transforming the agriculture sector based on agroecosystem and vulnerability assessment through: (1) establishing voluntary local institutions (IP); (2) accelerating access to technology and investment; (3) improving market mechanisms for climate resilient and sustainable products and practices; (4) defining environmental management as a community-level issue; and (5) empowering community members to address governance matters at the local level, and empowering communities to identify broader governance needs. 

## 6. Conclusions and Recommendations

Climate change adaptation in agricultural communities—and, for that matter, agriculture oriented development more broadly—is an enormously complex process. Discrepancies in adaptive capacity within and between communities are a product of ethnic interactions, government policies, family histories, and evolving natural resource conditions. Ability to adapt is also influenced by phenomena that lie far beyond a community’s control, including the rate of anthropogenic climate change, shocks to global commodity markets, and the cost of credit due to national and international economic dynamics. In this context, community-level adaptation analysis and interventions must be designed for local conditions while anticipating the need for flexibility in response to change at national and global scale.

This paper has presented an overview of findings from five years of climate impact and adaptation studies undertaken with communities of Choke Mountain. These communities represent a wide range of socioeconomic and biophysical conditions, such that the experiences of these studies can be scaled to other communities in the Ethiopian Highlands and, perhaps, beyond. 

The main conclusions and recommendations that result from an analysis of these studies and of three workshop discussions on the subject are the following.

- The natural resources that are the foundation for the economy of the country are under intense pressure from population growth and inappropriate traditional farming and management practices. The quality of land management has become an increasing matter of concern due to the additional stresses of climate change and population growth. Although there are many approaches used, *there remains considerable scope to further develop a clear conceptual framework and set of guidelines necessary to understand and measure vulnerability and to develop feasible adaptation strategies*.
- The Choke Mountain watersheds are characterized by considerable biophysical and socio-economic diversity. Biophysical diversity in micro-climate, soil properties, vegetation types and water resources results in high spatial variability in climate impacts and associated response measures. Socio-economic diversity adds an additional set of considerations relevant to vulnerability and adaptation options. The ecosystem and livelihood framework applied at Choke Mountain is a useful tool for planning, monitoring and evaluation of climate impacts and adaptation strategies. *It provides a method for advancing resilience against a baseline and for monitoring incremental change towards defined resilience outcomes, as established and evaluated by a diverse set of stakeholders*. In this regard, the Choke Mountain study can be a model for development-relevant research in the context of climate change.
- In the Blue Nile Highlands, understanding of climate change, both its manifestation and its impacts, is limited to a few experts and decision-makers. Therefore, *there is a need for a carefully conceived, evidence-based communication process that will enhance awareness of climate change and available response measures*, for both policy makers and those involved in community-level development.
- Informed adaptation strategies must take into account available scientific data on climate change impacts and also the vulnerability and perceptions of stakeholders; at present, information on both physical and social aspects of climate adaptation is limited, as are strategies for adapting in the context of uncertainty within the climate resilience framework. To address this limitation, *integrated research on both physical science and adaptive capacity is required*: the design and implementation of adaptation measures should include not only technical aspects, but also the transformation of the socio-cultural patterns that link them to the environment.
- Due to a lack of information on the impacts of climate variability and climate change on ecosystems and community livelihoods, and due to our limited knowledge of socioecological dynamics, *there are high levels of uncertainty regarding future projections and system responses*. Such constraints could limit adaptation responses.
- Research results from Choke Mountain and elsewhere indicate that *participatory technology development and implementation centers, established with broad participation from local stakeholders and national and international experts, offer an opportunity for effective and efficient technology development, uptake and dissemination*. This model is being applied in the form of Innovation Platforms (IP) for the Ethiopian highlands.
- *The major challenge is to translate the climate resilience framework into an effective, accountable, and democratic planning and negotiation process*. In many parts of the country, decisions on land conservation and investments in productivity and sustainability of ecosystems are made by local administrators without the involvement of local communities. Since the participation of farmers is vital for the sustainability of land management, farmer priorities must be taken into account. Research on climate resilience should include farmer participation to identify methods for improving land productivity and return on their labour. This conclusion is consistent with the findings of a recent IUCN review of econsystem-based adaptation studies across several countries [27].
- Due to the aforementioned uncertainty, *the design of adaptation measures must include a monitoring strategy* that provides information about the effectiveness of all adaptation measures.
- *The effective promotion of access to information on vulnerabilities, impacts and adaptation to climate change represents an indispensable element in building Ethiopia’s national response to climate change*. Effective information dissemination and networking (in the right format, quality and language) enhances stakeholders’ knowledge base for proactive engagement on climate change and its effects, and creates a sound foundation for policy formulation and action on climate change adaptation.

These conclusions are presently being integrated to climate resilience initiatives in the Blue Nile/Abay Highlands, most notably through the establishment of Innovation Platforms in support of a Climate Resilient and Green Economy. Experiences with these IP will provide a continuously growing body of knowledge and analysis to support the design of climate resilience building activities in the Ethiopian Highlands and in other agricultural economies striving to build resilience in an evolving climate.
